# Evolution of Bordetellae from Environmental Microbes to Human Respiratory Pathogens: Amoebae as a Missing Link

**DOI:** 10.3389/fcimb.2017.00510

**Published:** 2017-12-11

**Authors:** Dawn L. Taylor-Mulneix, Illiassou Hamidou Soumana, Bodo Linz, Eric T. Harvill

**Affiliations:** Department of Infectious Diseases, Center for Vaccines and Immunology, College of Veterinary Medicine, University of Georgia, Athens, GA, United States

**Keywords:** *Bordetella*, Amoeba, *Dictyostelium discoideum*, environmental microbes, respiratory pathogens

## Abstract

The genus *Bordetella* comprises several bacterial species that colonize the respiratory tract of mammals. It includes *B. pertussis*, a human-restricted pathogen that is the causative agent of Whooping Cough. In contrast, the closely related species *B. bronchiseptica* colonizes a broad range of animals as well as immunocompromised humans. Recent metagenomic studies have identified known and novel bordetellae isolated from different environmental sources, providing a new perspective on their natural history. Using phylogenetic analysis, we have shown that human and animal pathogenic bordetellae have most likely evolved from ancestors that originated from soil and water. Our recent study found that *B. bronchiseptica* can evade amoebic predation and utilize *Dictyostelium discoideum* as an expansion and transmission vector, which suggests that the evolutionary pressure to evade the amoebic predator enabled the rise of bordetellae as respiratory pathogens. Interactions with amoeba may represent the starting point for bacterial adaptation to eukaryotic cells. However, as bacteria evolve and adapt to a novel host, they can become specialized and restricted to a specific host. *B. pertussis* is known to colonize and cause infection only in humans, and this specialization to a closed human-to-human lifecycle has involved genome reduction and the loss of ability to utilize amoeba as an environmental reservoir. The discoveries from studying the interaction of *Bordetella* species with amoeba will elicit a better understanding of the evolutionary history of these and other important human pathogens.

## Introduction

The genus *Bordetella* comprises several bacterial species, which are pathogenic to animals and humans. The most clinically relevant species is *B. pertussis*, the causative agent of Pertussis disease, or Whooping Cough. This acute respiratory disease, known for the characteristic symptoms of paroxysmal cough, whooping, and post-tussive vomiting, is particularly serious and sometimes fatal in infants and elderly people. From a veterinary perspective *B. bronchiseptica* and *B. avium* are important. *B. bronchiseptica*, a respiratory pathogen of a wide range of mammals, causes a variety of pathologies ranging from chronic and often asymptomatic infection to more severe and acute diseases, including bronchopneumonia and atrophic rhinitis in pigs, bronchitis in cats, snuffles in rabbits, and acute tracheobronchitis (“Kennel Cough”) in dogs (Goodnow, 1980; Mattoo and Cherry, 2005). *B. avium* infects the respiratory tract of wild and domesticated birds, particularly turkeys, causing a respiratory disease with the symptoms known as bordetellosis or coryza in turkey chicks (Panigrahy et al., 1981; Kersters et al., 1984; Raffel et al., 2002). Recently, we identified that the bordetellae likely originated evolutionarily from soil and water environments and showed that the animal pathogen, *B. bronchiseptica*, can utilize amoeba, such as *Dictyostelium discoideum*, as environmental reservoirs and transmission vectors. We hypothesize that evolving the ability to evade amoebic predation and utilize amoebae as an environmental niche allowed bordetellae the transition from survival in soil and water to being respiratory pathogens.

## Environmental origin of *Bordetella*

Bacteria of the genus *Bordetella* are known as colonizers of human and animal respiratory tracts. *B. pertussis, B. bronchiseptica*, and *B. parapertussis* form an evolutionary monophyletic group commonly referred to as “classical” bordetellae. Over the past decades, more distantly related *Bordetella* species, the “non-classical” species, have been described that colonize the respiratory tract and cause disease in birds (*B. avium* and *B. hinzii*), mice (*B. pseudohinzii*) and humans (*B. holmesii, B. bronchialis, B. flabilis*, and *B. sputigena*) (Kersters et al., 1984; Vandamme et al., 1995, 2015; Weyant et al., 1995; Ivanov et al., 2015, 2016), or were isolated from infected wounds of immunocompromised patients (*B. trematum* and *B. ansorpii*) (Vandamme et al., 1996; Ko et al., 2005). Although species associated with humans and animals have attracted the most attention, recent studies have revealed *Bordetella*-like bacteria in the environment. *B. petrii*, the first *Bordetella* species identified from a non-animal source, was initially isolated from an anaerobic bioreactor culture enriched by river sediment (von Wintzingerode et al., 2001), and has been subsequently found in marine sponges (Sfanos et al., 2005) and grass root consortia (Chowdhury et al., 2007). *B. petrii* has also been isolated from immunocompromised patients with ear infections and with pulmonary disease (Fry et al., 2005; Biederman et al., 2015; Nagata et al., 2015), suggesting that this species could also be an opportunistic pathogen in humans and animals, as has been observed for *Pseudomonas* spp. (de Bentzmann and Plesiat, 2011). In a recent study, three novel environmental *Bordetella* species were described, *B. muralis, B. tumulicola*, and *B. tumbae*, that have been isolated from a plaster wall surface of 1,300-year-old mural paintings in Japan (Tazato et al., 2015). With the vastly improved ability to study bacterial communities inhabiting diverse environmental niches, including soil, rocks, water, air, ice, plants, and animals (Schuster, 2008; Blottiere et al., 2013; Cowan et al., 2015; Anantharaman et al., 2016; Gionfriddo et al., 2016; Makhalanyane et al., 2016), and the ability to identify sequences from *Bordetella*-like bacteria in metagenomics datasets, we expect to see an increase in the number of described environmental *Bordetella* species.

We recently identified numerous 16S rRNA sequences of bacteria isolated from various environmental sources, including soil, water, plants and sediment, that displayed high sequence similarity with the 16S rRNA gene sequence of *Bordetella* (Hamidou Soumana et al., 2017). Based on 48 full-length 16S RNA sequences of strains recovered from these environments, we performed a phylogenetic analysis to determine the relatedness between human- and animal-associated *Bordetella* species and those isolated from environmental samples. The phylogenetic tree provided evidence for an environmental origin of bordetellae, as sequences from environmental samples possessed a significantly higher genetic diversity than those from human- and animal-associated samples. Sequences from environmental samples were present in all 10 sequence clades, including sequence clades at the root of the phylogenetic tree. In contrast, sequences from animal-associated species were found in only four sequence clades at the top of the tree. Together, the order of branching events within the phylogenetic tree suggested that *Bordetella* species, including human-restricted pathogens, arose from environmental ancestors (Hamidou Soumana et al., 2017). The evolution and adaptation to human and animal hosts most likely occurred after acquisition of virulence factors that enabled them to respond to new hosts.

**Figure 1:**
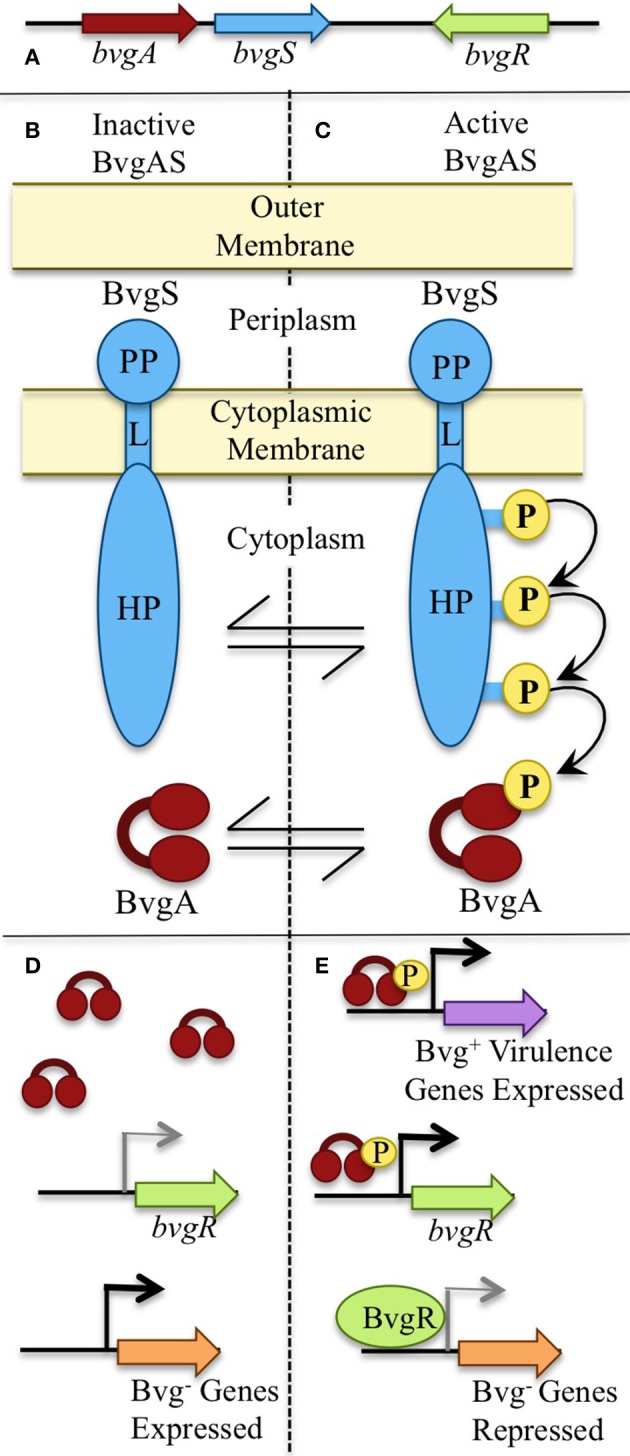
The BvgAS phosphorelay. **(A)** The master regulatory system of bordetellae, Bordetella Virulence Genes (BVG), is expressed by *bvgS* and *bvgA*. **(B,C)** BvgS is a transmembrane sensor protein consisting of a periplasmid domain (PP) connected to the histidine phosphotransfer domains (HP) in the cytosol through a linker domain (L). **(B)** BvgS is inactive and un-phosphorylated when bacteria grow at temperatures below 25°C. **(D)** Bvg^−^ phase genes are transcribed when the BvgAS system is inactive. **(C)** Upon receiving inducing signals such as 37°C, BvgS autophosphorylates and initiates a phosphor-relay that leads to phosphorylation and activation of BvgA. **(E)** When the BvgAS system is active, Bvg^+^ phase-associated genes are transcribed, including *bvgR*. BvgR represses expression of Bvg^−^ phase associated genes.

The ability of micro-organisms to adapt to different environments and hosts requires a high genomic plasticity coupled with the capacity to sense and respond to environmental changes (Aujoulat et al., 2012). Under laboratory conditions, classical bordetellae such as *B. bronchiseptica* can respond to different environmental stimuli by switching between two distinct life styles. When cultured at 37°C, which mimics mammalian host temperatures, expression of genes associated with virulence and colonization in mammalian hosts is up-regulated. However, *B. bronchiseptica* adopts a second life style when cultured at 25°C or lower, during which expression of virulence-associated genes is down-regulated while expression of a large, alternative set of genes, such as those involved in motility and growth in dilute nutrients, is up-regulated. Expression of the latter genes has been hypothesized to be important under extra-host growth conditions or in an environmental niche (Taylor-Mulneix et al., 2017). This global gene regulation is under the control of the two-component system BvgAS, consisting of a sensor protein, BvgS, a transcriptional activator, BvgA, and a transcriptional repressor, BvgR (Figure 1). Upon phosphorylation by BvgS, BvgA binds to the promoter regions of the Bvg-activated genes and activates transcription. The last gene, *bvgR*, is responsible for the regulation of the Bvg-repressed genes (Merkel et al., 1998). Virulence-associated factors are expressed in the Bvg positive (Bvg^+^) phase, and the alternative set of genes in the Bvg negative (Bvg^−^) phase (Figure 1). The ability to switch between life styles seems to be conserved amongst bordetellae as *bvgA* and *bvgS* gene homologs have been found in the genomes of animal-associated species as well as the environmental *B. petrii* (Gerlach et al., 2004; Gross et al., 2010; Linz et al., 2016). In addition, *B. petrii, B. bronchiseptica*, and *B. hinzii*, all known to associate with mammalian hosts, were shown to grow efficiently in soil at 25°C (Hamidou Soumana et al., 2017). These observations indicate that even though *B. bronchiseptica* has adapted to mammals, it has conserved the ability to survive under environment conditions, and respond to changes such as temperature fluctuations (Coote, 2001).

## Amoeba as an environmental reservoir of bordetellae

Recently, we have reported the novel ability of *B. bronchiseptica* strain RB50 to utilize amoebae as an environmental niche and transmission vector (Taylor-Mulneix et al., 2017). *B. bronchiseptica* was chosen for this study because it is believed to resemble the progenitor of the classical bordetellae which are most highly associated with human disease (Diavatopoulos et al., 2005). While *B. pertussis* and *B. parapertussis* have undergone genome reduction in their adaption to human, *B. bronchiseptica* retains the largest genome, the widest range of animal hosts, and characteristics like motility and nutrient scavenging that are associated with the ability to survive in some environmental reservoir. Indeed, our data found that *B. bronchiseptica* establishes a commensal relationship with amoeba. While, as of yet, there is no data indicating whether *B. bronchiseptica* results in any benefit or harm to the amoeba, there is clear evidence that it benefits from its interactions with the amoeba *Dictyostelium discoideum* (Taylor-Mulneix et al., 2017).

Amoeba predation involves chemotaxis toward their bacterial food source, internalization, phagocytosis, killing, and metabolizing bacteria. *B. bronchiseptica* is amongst the few bacteria that can survive intracellularly within amoeba for extended periods of time (Hägele et al., 2000; Abd et al., 2003; Greub and Raoult, 2004). This intracellular survival was observed both in *D. discoideum* and in *Acanthamoeba castellanii* (Taylor-Mulneix et al., 2017). Furthermore, we showed that *B. bronchiseptica* can localize to the amoeba sori and associate with the amoeba through multiple passages while utilizing another bacterial food source. These data were quite striking as association with the amoeba was maintained through multiple passages despite the observation that *B. bronchiseptica* localized to the sori are not intracellular within the *D. discoideum* spores. However, *B. bronchiseptica* within the amoeba sori up-regulate expression of factors associated with cell adherence. These data suggest that bordetellae have evolved mechanisms that allow them to survive in long-term association with amoeba, including surviving intracellularly within these phagocytic cells. These mechanisms may also allow them to evade phagocytosis and killing by mammalian immune cells (Mattoo et al., 2000).

As the BvgAS two-component system appears to regulate genes associated with the different life styles of bordetellae (Bvg^+^ in animal hosts and Bvg^−^ in a putative environmental niche), we hypothesized that the Bvg^−^ phase may be important during the bacterial interaction with amoebae. Indeed, *B. bronchiseptica* that were mutated to constantly express Bvg^+^ associated virulence factors had a significantly lower recovery from the amoeba sori in comparison to wildtype or a Bvg^−^ mutant. These observations indicated an advantage for the Bvg^−^ phase and represents the first report of a role for the Bvg^−^ phase in association with any host. Therefore, the presence of the Bvg^−^ phase across the *Bordetella* genus may be a clue to this genus' origin and evolution (Taylor-Mulneix et al., 2017).

## Amoeba as a “training ground” for human pathogens

The evolution of *Bordetella* species from environmental microbes to animal and human pathogens suggests the existence of an intermediate stage, or commensal host, that has facilitated the adaptation. Free-living amoeba such as *Acanthamoeba* spp. have been shown to serve as an environmental niche for several opportunistic bacterial pathogens such as *Burkholderia* spp., *Pseudomonas aeruginosa, Listeria monocytogenes, Legionella pneumophila*, and *Mycobacterium* (Cirillo et al., 1997; Hägele et al., 2000; Greub and Raoult, 2004; Drancourt, 2014; DiSalvo et al., 2015; Jose Maschio et al., 2015). *L. pneumophila* is an example of a bacterial pathogen that utilizes a similar strategy of invasion and life style inside both amoeba and macrophages (Hägele et al., 2000; Greub and Raoult, 2004). Our research has now added *B. bronchiseptica* to the list of bacteria that can survive amoebic predation and utilize amoeba as an environmental reservoir. Interestingly, the ability of *B. bronchiseptica* to survive internalization and resist digestion by eukaryotic cells is not limited to amoebae. Upon infection of a mammalian host, *B. bronchiseptica* and *B. pertussis* can survive inside macrophages, thereby enabling the bacteria to evade host immunity (Siciliano et al., 2006; Lamberti et al., 2010, 2013). The strategy to hide inside host immune cells such as macrophages provides a means of persistence in the host and facilitates the spread to other tissues and organs.

Single-celled amoeba and macrophages are fairly similar physiologically, therefore, these observations suggest that adaptation of environmental bacteria to amoeba may have served as an intermediate step to become animal- or human-associated pathogens (Molmeret et al., 2005). Similar to other species, the long-term bacteria-amoeba association may be the key that has allowed bordetellae to evolve the ability to associate with vertebrate hosts.

## Genome reduction results in failure to employ amoeba as a host

*B. pertussis* and *B. parapertussis* evolved independently from a *B. bronchiseptica*-like ancestor (Parkhill et al., 2003; Diavatopoulos et al., 2005; Park et al., 2012; Linz et al., 2016). These species possess ≥98% nucleotide identity on the genome level and share many important virulence-associated factors, including toxins and adhesins, suggesting a relatively recent divergence. Indeed, a global phylogeny based on whole genome sequences of a worldwide collection of *B. pertussis* isolates showed two deep branches that coalesce to a last common ancestor about 2,300 (range 1,428–3,340) years ago. A subsequent expansion and diversification of one of the branches, that contains over 98% of all isolates, occurred about 500 years ago (Bart et al., 2014), which correlates with the first historic descriptions of Whooping Cough disease in Persia (Aslanabadi et al., 2015) and Europe (Bart et al., 2014). The extremely low sequence diversity in *B. pertussis* (Linz et al., 2016) indicates that the speciation and specialization to the human host was associated with a strong genetic bottleneck that drastically reduced the sequence diversity. It appears that this process was accompanied by the acquisition and massive expansion of insertion sequence elements (ISE), particularly of IS*481*. ISE's were inserted at hundreds of genomic locations, and homologous recombination between these identical DNA repeats resulted in a mosaic-like structure of the chromosome, in which short blocks of perfect collinearity are broken up by nearly 150 individual rearrangements, including chromosomal inversions. In fact, 88% of the genomic rearrangements in the genome of *B. pertussis* strain Tohama_I, in comparison to the genome of *B. bronchiseptica* strain RB50, are bordered by IS elements, mostly IS*481* (Parkhill et al., 2003). Recombination between ISE's also caused a large amount of deletion in the chromosome, which is reflected in the much smaller genome of *B. pertussis* (about 4.1 Mb with 3,816 CDS's) compared to that of *B. bronchiseptica* (about 5.3 Mb with 5,007 CDS's). *B. pertussis* lost over 1,000 genes during the evolution from a *B. bronchiseptica*-like ancestor, most of which encode transcriptional regulators, proteins involved in transport and metabolism of a wide range of compounds, as well as proteins of unknown function (Linz et al., 2016).

Similar to *B. pertussis, B. parapertussis* has also undergone host specialization; one lineage causes pertussis-like disease in humans (hereafter referred to as *Bpp*_hu_), while the other causes pneumonia in sheep (*Bpp*_ov_). Both *B. parapertussis* lineages have undergone genome reduction *via* ISE's, but much less drastic than *B. pertussis*, resulting in genome sizes of 4.7 Mb in *Bpp*_hu_ strain 12822 and of 4.8 Mb in *Bpp*_ov_ strain Bpp5 (Parkhill et al., 2003; Park et al., 2012). Genome comparisons of *Bpp*_hu_ strain 12822 and of *Bpp*_ov_ strain Bpp5 to *B. bronchiseptica* strain RB50 revealed a large degree of collinearity between the genomes, with a limited number of *IS1001*-flanked genomic breakpoints. However, the evolution of both *B. parapertussis* lineages proceeded independently as evidenced by different genomic rearrangements and different gene content (Parkhill et al., 2003; Park et al., 2012; Linz et al., 2016). As a result, 3,592 genes are shared between the *B. parapertussis* genomes while 829 genes are specific to the genome of *Bpp*_hu_ strain 12822 and 592 are specific to the genome of *Bpp*_ov_ strain Bpp5.

Another human-restricted pathogen, *B. holmesii*, which has been co-isolated with *B. pertussis* during outbreaks of Whooping Cough, also appears to have undergone substantial genome reduction. The genome of this emerging non-classical species (3.7 Mb) is about 1.2 Mb smaller than that of its closest relative *B. hinzii* (4.9 Mb). The *B. holmesii* genome contains multiple copies of three different ISE's (IS*407*, IS*481*, and IS*L3*). Similar to *B. pertussis*, many of the putative genomic breakpoints that disrupt the genome synteny are flanked by ISE's, suggesting genome reduction and rearrangements through homologous recombination between ISE's (Linz et al., 2016). Thus, specialization of several *Bordetella* species to an exclusively human host was associated with acquisition and expansion of different classes of ISE's, and subsequent recombination between those perfect DNA repeats resulted in chromosomal rearrangements and extensive genome reduction in each of those lineages.

**Figure 2:**
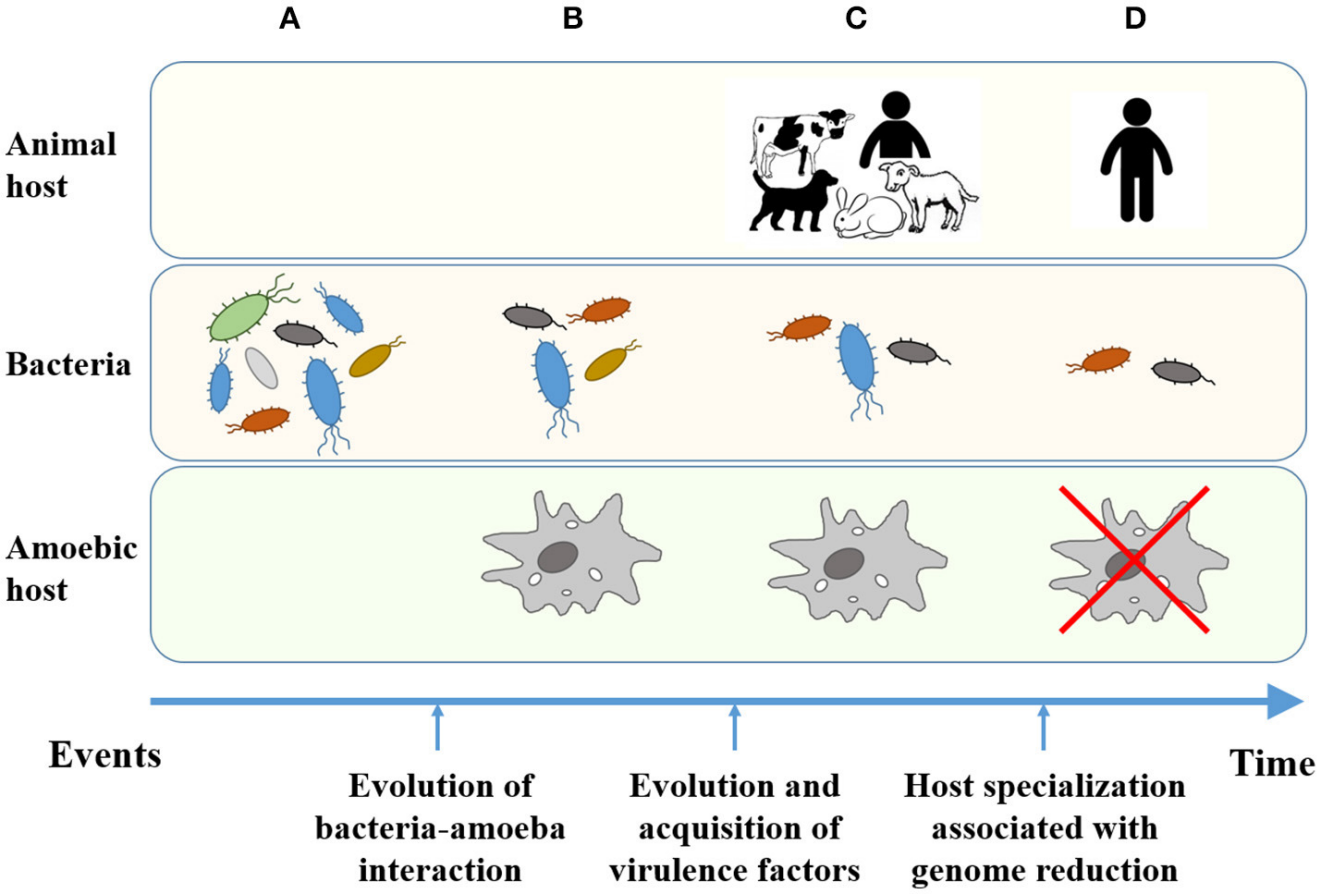
Evolution of bacteria from environmental microbes to human-restricted pathogens. **(A)** Environmental bacteria as a food source for amoebae. **(B)** Bacteria developed resistance to digestion and the ability to interact with the new eukaryotic host. **(C)** Bacteria able to interact with and utilize amoebae evolved to animal pathogens. **(D)** Host-specialized bacterial pathogens lost the ability to resist predation and interact with lower eukaryotes.

During the assessment of the ability of classical bordetellae to utilize *D. discoideum*, an interesting dichotomy has arisen. The ability to survive intracellularly and localize to the amoeba sori is conserved amongst *B. bronchiseptica*, but is absent in *B. pertussis* (manuscript in preparation). These data suggested that as *B. pertussis* adapted to the closed-cycle of using only humans as a host it underwent genome reduction and lost the ability to utilize *D. discoideum* as an environmental reservoir (Figure 2). In support of this theory, the *Bpp*_ov_ strain Bpp5 retained the ability to survive intracellularly and localize to the sori, while the human isolated *Bpp*_hu_ strain 12822 did not (manuscript in preparation). The observed difference between the two *B. parapertussis* lineages may be associated with a substantially different gene content in their genomes. All in all, the data suggest that while the ancestor-like *B. bronchiseptica* strains can utilize the amoeba as an environmental reservoir, genome reduction associated with host specialization has led to the inability of human-adapted bordetellae to evade amoebic predation (Figure 2). While the ability of the human-associated *B. holmesii* to employ the amoebic life cycle remains to be elucidated, we predict that its severe genome reduction likely involved loss of genes necessary for survival in and utilization of amoebae. Thus, during the evolution of bordetellae from soil microbes to human pathogens, the interaction with and role of amoeba is an important step that may be overlooked if one were to only compare *B. pertussis* with soil isolated bordetellae. All in all, it remains important to consider the whole bordetellae genus and the ability of each individual species to interact with amoeba. The association of genome data with the ecology of amoebic-bacterial interaction will provide important clues that will ultimately reveal key steps in the evolution of *Bordetella* from environmental microbes to human pathogens. Along this line, we have begun to investigate the interaction of multiple *Bordetella* species with the social amoeba *D. discoideum* to expand this fascinating field of research beyond the classical bordetellae.

## Conclusion

The human immune system is so complex that it is able to efficiently resist invasion and/or resolve infection with little damage to the host. Yet, many human pathogens have arisen from progenitors found in soil and water environments. Therefore, it is important to understand the evolutionary pressures, which allow bacteria adapted to success in other environments to emerge as important human pathogens that cause major disease burden. Herein, we describe that the interaction of bordetellae with predatory amoeba in the environment may have favored the evolution of tools that prepared them for their interactions with mammalian phagocytes, contributing to their emergence as important human pathogens. As these bacteria established a chain of transmission in mammals and further adapted to these hosts, their specialization, and genome reduction, may come with a cost of losing the environmental reservoir. Further work in studying these and other human pathogens and their interaction with amoebae will be important for understanding the mechanisms by which pathogens can evolve and develop mechanisms to evade host immune systems.

## Author contributions

DT-M, IH, and BL contributed equally to this manuscript in regards to concept, writing, and editing. These authors should all be considered equally contributing first authors and we wish that the publication would reflect this. EH contributed to editing and obtaining funding.

### Conflict of interest statement

The authors declare that the research was conducted in the absence of any commercial or financial relationships that could be construed as a potential conflict of interest.
